# Doublet Versus Single Agent as Second-Line Treatment for Advanced Gastric Cancer: A Meta-Analysis of 10 Randomized Controlled Trials

**DOI:** 10.1097/MD.0000000000002792

**Published:** 2016-03-03

**Authors:** Yong Zhang, Bing Ma, Xiao-Tian Huang, Yan-Song Li, Yu Wang, Zhou-Lu Liu

**Affiliations:** From the Department of General Surgery, General Hospital of Chinese People's Liberation Army, Beijing, China.

## Abstract

The purpose of this study was to perform a meta-analysis of randomized controlled trials (RCTs) to compare the efficacy and safety of doublet versus single agent as second-line treatment for advanced gastric cancer (AGC).

A comprehensive literature search was performed to identify relevant RCTs. All clinical studies were independently identified by 2 authors for inclusion. Demographic data, treatment regimens, objective response rate (ORR), and progression-free survival (PFS) and overall survival (OS) were extracted and analyzed using Comprehensive Meta-Analysis software (Version 2.0).

Ten RCTs involving 1698 pretreated AGC patients were ultimately identified. The pooled results demonstrated that doublet combination therapy as second-line treatment for AGC significantly improved OS (hazard ratio [HR] 0.87, 95% confidence interval [CI]: 0.78–0.97, *P* = 0.011), PFS (HR 0.79, 95% CI: 0.72–0.87, *P* < 0.001), and ORR (relative risk [RR] 1.57, 95% CI: 1.27–1.95, *P* < 0.001). Sub-group analysis according to treatment regimens also showed that targeted agent plus chemotherapy significantly improve OS, PFS, and ORR. However, no significant survival benefits had been observed in doublet cytotoxic chemotherapy when compared with single cytotoxic agent. Additionally, more incidences of grade 3 or 4 myelosuppression toxicities, diarrhea, and fatigue were observed in doublet combination groups, while equivalent frequencies of grade 3 or 4 thrombocytopenia and nausea were found between the 2 groups.

In comparison with single cytotoxic agent alone, the addition of targeted agent to mono-chemotherapy as salvage treatment for pretreated AGC patients provide substantial survival benefits, while no significant survival benefits were observed in doublet cytotoxic chemotherapy regimens.

## INTRODUCTION

Gastric cancer is the fourth most common malignant disease and the second leading cause of cancer mortality worldwide, accounting for 8% (989,600 million) of the total new cancer cases and 10% (738,000) of the total cancer deaths in 2008.^1^ Despite the advances in diagnostic techniques, surgery, and adjuvant treatment, nearly 50% of patients with locally advanced-stage gastric cancer relapse after gastrectomy.^2,3^ For such patients, palliative chemotherapy is the mainstay treatment to prolong the survival. Currently, systematic chemotherapy based on 5-fluoropyrimidines/platinum plus human epidermal growth factor receptor-2 antibody or not is recognized as the standard first-line chemotherapy. However, the efficacy of first-line chemotherapy is modest with median survival 8 to 12 months,^4–6^ and most patients are nonresponders or eventually experience disease progression. After first-line treatment of gastric cancer, no standard second-line treatment is yet approved by the US Food and Drug Administration, or other governmental drug regulatory agencies. Until now, mono-chemotherapy using taxanes or irinotecan is the commonly used regimen for advanced gastric cancer (AGC) patients who fail to first-line chemotherapy. A recent meta-analysis of 3 randomized controlled trials (RCTs) conducted by Kim et al found that salvage mono-chemotherapy using docetaxel or irinotecan significantly decreased the risk of death (hazard ratio [HR] 0.64, 95% confidence interval [CI]: 0.52–0.79, *P* < 0.001) when compared with supportive cancer treatment,^7^ which has been confirmed by 2 subsequent meta-analyses.^8,9^ In an attempt to improve treatment outcomes, combination regimens as salvage treatment have been investigated in several RCTs, but the results are controversial. Therefore, we perform a systematic review and meta-analysis of all available RCTs to compare the efficacy and safety of doublet versus single agent as second-line chemotherapy for AGC patients.

## MATERIALS AND METHODS

### Study Design

We conducted this meta-analysis adheres to the Preferred Reporting Items for Systematic Reviews and Meta-Analyses (PRISMA) statements (Supplemental Table 1).^10^ This study did not involve human subjects, so informed consent was not required. In addition, no approval was required from any institutional review board.

### Identification and Selection of Studies

We conducted an independent review of related studies from 4 databases, including Embase, Medline, the Cochrane Central Register of Controlled Trials, and the Cochrane Database of Systematic Reviews, from the date of inception of every database to August 2015. The keywords were “gastric cancer,” “gastric carcinoma,” “gastric neoplasm,” “previously treated,” “refractory,” “salvage treatment,” and “RCTs.” Additional references were searched through manual searches of the reference lists and specialist journals.

Two investigators (YZ and BM) independently assessed the eligibility of trials. Clinical trials that met the following criteria were included: prospective RCTs involving previously treated AGC; trials comparing doublet combinations with single cytotoxic agent; the included studies had sufficient data for extraction. The decision to include trials for analysis in this study was reached by consensus. We also ask the principal investigators of potential trials to provide missing data and updates by electronic mail. The Jadad scale was used to assess the quality of the included trials based on the reporting of the studies’ methods and results.^11^

### Data Extraction and Outcome Measures

Data abstraction was conducted independently by 2 investigators (YZ and BM). A third investigator (Z-LL) reviewed all data entries. For each study, the following data were extracted: first author's name, year of publication, median age, treatment regimens, number of enrolled patients, median progression-free survival (PFS), median overall survival (OS), and grade 3/4 toxicities. A standardized excel file was used for data extraction. The primary outcome of this study is OS. Secondary outcomes included PFS, objective response rate (ORR), and grade 3 to 4 toxicities.

### Statistical Analysis

Statistical analysis was performed using Comprehensive Meta-Analysis Version 2 software (Biostat, Englewood, NJ). Between-study heterogeneity was estimated using the χ^2^-based Q statistic.^12^ Heterogeneity was considered statistically significant when *P*_heterogeneity_ < 0.05 or I^2^ > 50%. Meta-analysis was performed using a random-effects model when significant heterogeneity was found among the trials. The results were reported as HRs with 95% CIs for OS and PFS analyses. Pooled relative risks (RRs) with 95% CIs were used for ORR and toxicities. The main modality of presenting numerical data in visual form was the forest plot. We also performed sub-group analysis according to treatment regimens or cytotoxic agents. The presence of publication bias was evaluated by using the Begg and Egger tests.^13,14^ All *P* values were 2-sided. All CIs had a 2-sided probability coverage of 95%.

## RESULTS

### Selection of Included Studies

**FIGURE 1:**
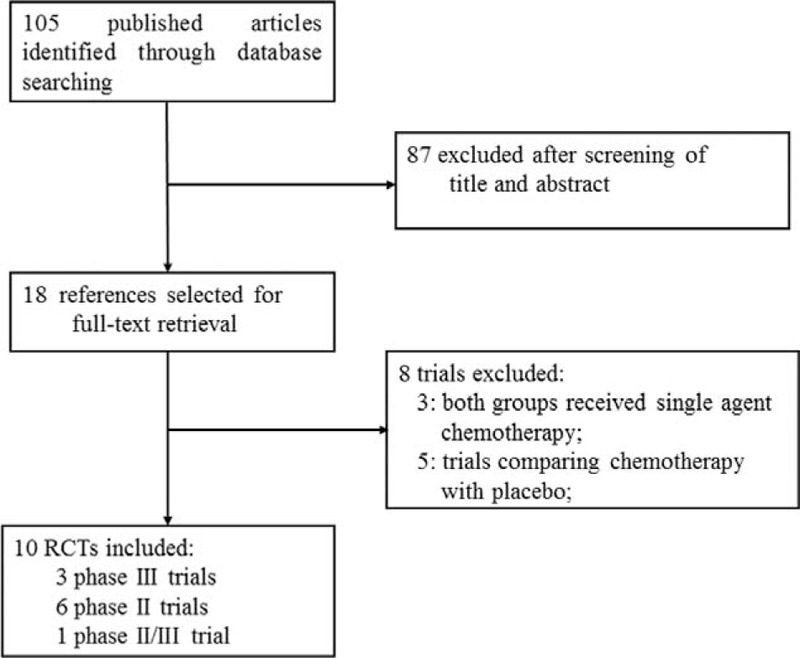
Selection process for clinical trials included in the meta-analysis.

A total of 105 related publications were identified from the database search. In the initial screening, a total of 18 potentially relevant trials were selected for full-text retrieval. After reading the content of full paper, a total of 10 RCTs that met the inclusion criteria were included in the present study (Figure 1).

### Study Characteristics

Table 1 shows the baseline characteristics of all relevant trials. The total number of enrolled patients in each study ranged from 24 to 665, with a total of 1698 patients. According to the inclusion criteria of each trial, patients were required to have an adequate renal, hepatic, and hematologic function. Each of the RCTs that satisfied the inclusion criteria comparing doublet combination group versus single cytotoxic chemotherapy. The quality of each included study was roughly assessed using Jadad score, 1 trial was a double blinded placebo-controlled trials, thus had Jadad score of 5,^15^ and 4 trials, which did not mention the concealment of allocation in the randomization process, thus had Jadad scores of 3,^16–19^ and the other 5 trials had Jadad score of 2.^20–24^

**TABLE 1:**
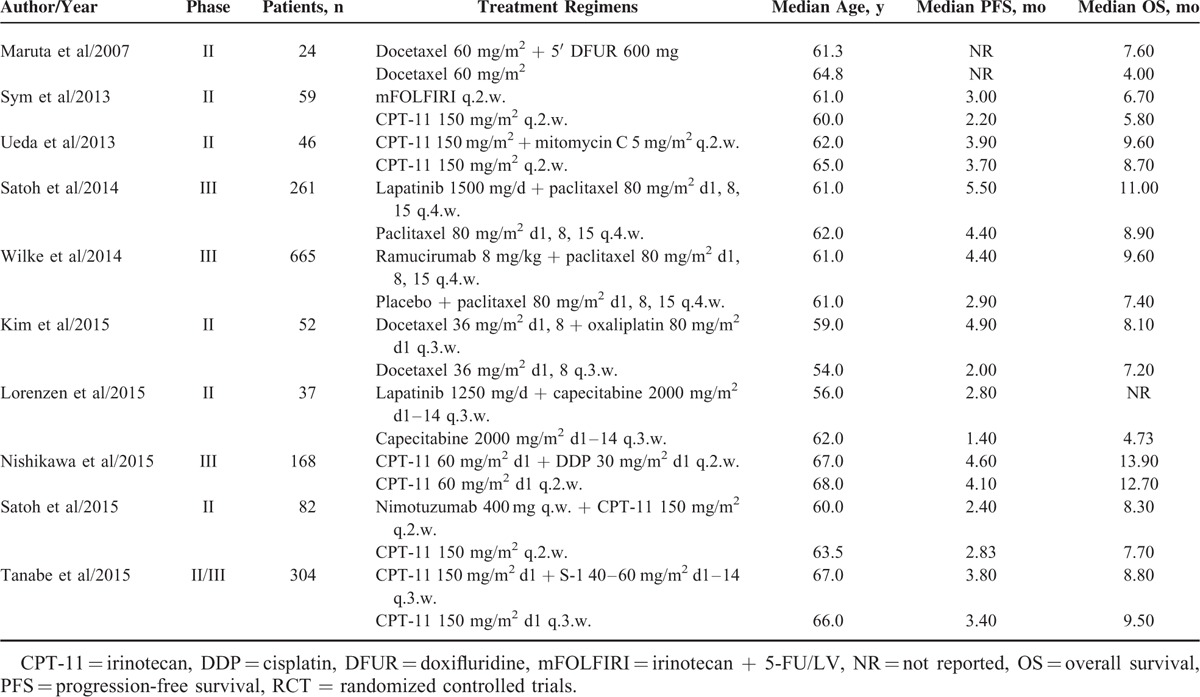
Baseline Characteristics of 10 RCTs for Meta-Analysis

### Overall Survival

**FIGURE 2:**
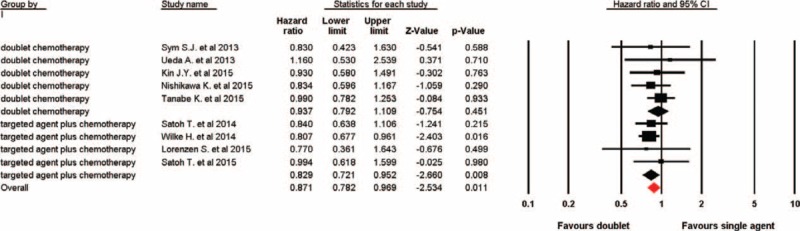
Fixed-effects model of hazard ratio (95% CI) of overall survival associated with doublet versus single agent. CI = confidence interval.

Nine of the 10 trials reported OS in the study patients. There was a significant improvement in OS in favor of doublet combination therapy compared with single cytotoxic agent (HR 0.87, 95% CI: 0.78–0.97, *P* = 0.011, Figure 2) using a fixed-effects model (I^2^ = 0, *P* = 0.93). Sub-group analyses based on treatment regimens also showed that targeted agent plus cytotoxic chemotherapy (HR 0.83; 95% CI: 0.72–0.95, *P* = 0.008) significantly improved OS, but not for doublet cytotoxic chemotherapy (HR 0.94; 95% CI: 0.79–1.11, *P* = 0.45, Figure 2). Considering type of combination therapy used, we found that the improvement in OS was more pronounced in the trials with taxanes-based doublet therapy (HR 0.83, 95% CI: 0.72–0.95, *P* = 0.007) while no difference was observed in studies with CPT-11 (irinotecan)-based doublet therapy (HR 0.95, 95% CI: 0.80–1.14, *P* = 0.59).

### Progression-Free Survival

**FIGURE 3:**
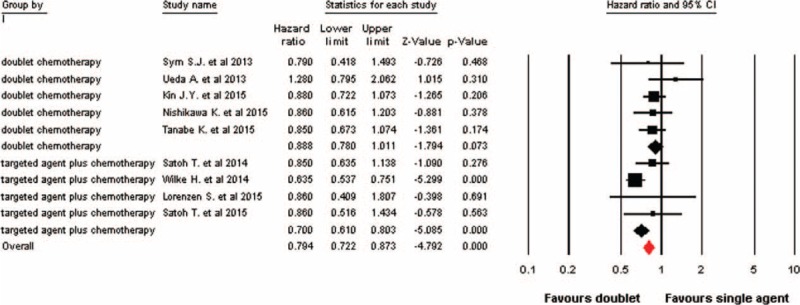
Fixed-effects model of hazard ratio (95% CI) of progression-free survival associated with doublet versus single agent. CI = confidence interval.

Data on PFS were available in 9 trials. Using a fixed-effects model (I^2^ = 36.4%, *P* = 0.13), doublet combinations significantly improve PFS giving HR 0.79 (95% CI: 0.72–0.87, *P* < 0.001, Figure 3) compared with single cytotoxic agent. The significant improvement in PFS was also significant in studies comparing targeted agent plus chemotherapy with single cytotoxic agent (HR 0.70; 95% CI: 0.61–0.80, *P* < 0.001), while a tendency to improve PFS was observed in doublet cytotoxic chemotherapy trials (HR 0.89; 95% CI: 0.78–1.01, *P* = 0.073, Figure 3). Similarly, we also found that taxanes-based doublet therapy significantly improved PFS (HR 0.77, 95% CI: 0.61–0.97, *P* = 0.026) when compared taxanes alone, while on difference was observed in studies with CPT-11-based doublet combination (HR 0.89, 95% CI: 0.76–1.05, *P* = 0.16).

### Overall Response Rate

**FIGURE 4:**
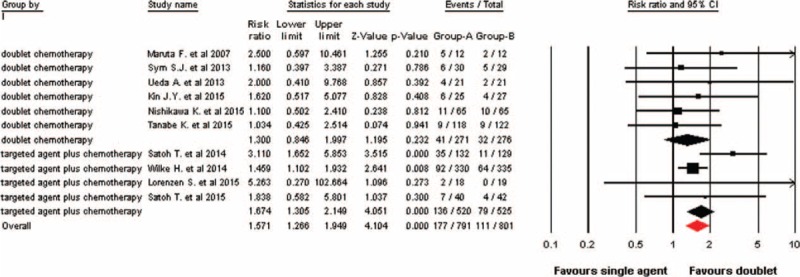
Fixed-effects model of relative risk (95% CI) of objective response rate associated with doublet versus single agent. CI = confidence interval.

A total of 10 trials were included for ORR analysis, and the pooled analysis using a fixed-effects model (I^2^ = 0%, *P* = 0.54) indicated that doublet combination therapy significantly improved ORR in comparison with single agent (RR 1.57, 95% CI: 1.27–1.95, *P* < 0.001, Figure 4). In sub-group analysis according to treatment regimen, we also found that targeted agent plus chemotherapy significantly improve ORR (RR 1.67; 95% CI: 1.31–2.15, *P* < 0.001), but not for doublet cytotoxic chemotherapy (RR 1.30; 95% CI: 0.85–2.00, *P* = 0.232, Figure 4). Moreover, we also carried out a sub-group analysis according to cytotoxic agents and indicated that the addition of agents to taxanes-based therapy significantly improved ORR (RR 1.67, 95% CI: 1.31–2.14, *P* < 0.001) when compared with taxanes alone, while no significant benefit of ORR was found in trials using CPT-11-based doublet therapy (RR 1.24, 95% CI: 0.79–1.95, *P* = 0.35).

### Safety

Nine trials presented data on hematologic toxicities (anemia, leukopenia, and neutropenia), 6 trials on thrombocytopenia, 10 on diarrhea, 7 on nausea, and 8 on fatigue. Table 2 shows the overall occurrence of high-grade (≥grade 3) toxic effects with doublet combination agents versus single cytotoxic agent. There were significantly more incidences of grade 3 or 4 hematologic toxicities (anemia, leukopenia, and neutropenia), diarrhea (RR 1.70, 95% CI: 1.03–2.83, *P* = 0.04), fatigue (RR 1.84, 95% CI: 1.21–2.80, *P* = 0.004) in doublet combination groups compared with single agent group, while equivalent frequencies of grade 3 or 4 thrombocytopenia (RR 0.62, 95% CI: 0.11–3.39, *P* = 0.58) and nausea (RR 0.87, 95% CI: 0.53–1.42, *P* = 0.57) were found between the 2 groups.

**TABLE 2:**
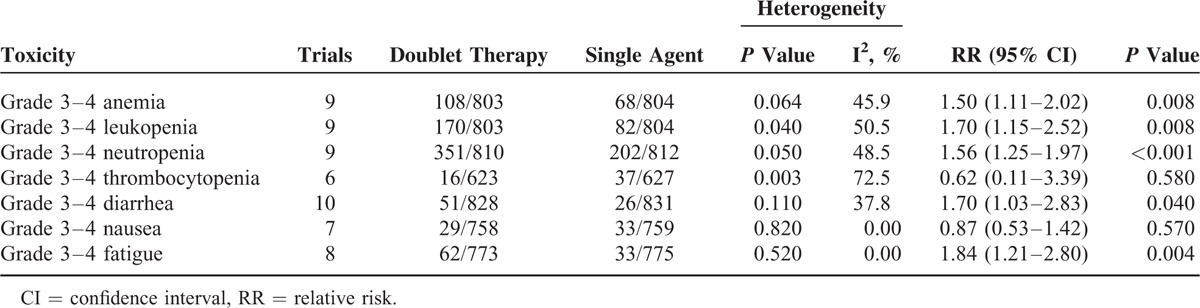
Outcome of Grade 3 or 4 Toxicity Comparing Doublet Versus Single Agent

### Publication Bias

Begg funnel plot and Egger test were performed to assess the publication bias of literatures. No publication bias was detected for the primary endpoint of this study (HR of OS) by the funnel plot, Begg test (*P* = 0.46), and Egger test (*P* = 0.39). Similarly, no publication bias was detected for the secondary endpoint of this study by Begg test (*P* = 0.47 for PFS and *P* = 0.07 for ORR) and Egger test (*P* = 0.16 for PFS and *P* = 0.49 for ORR).

## DISCUSSION

Until recently, treatment therapies for previously treated AGC patients are still limited, with moderate efficacy. Mono-chemotherapy using taxanes or irinotecan is the commonly drugs for AGC patients in this setting. Due to the aggressive and rapid fatal disease course of AGC, the development of systematic chemotherapy using combinations of agents, such as novel cytotoxic agents or targeted agents, is rational for the salvage treatment of this disease, especially in those patients with good performance status. In fact, several RCTs have been conducted to assess the efficacy and safety of doublet versus single agent as second-line therapy for AGC patients, but the results are controversial. As a result, we conduct this systematic review and meta-analysis to compare the efficacy and toxicities of combination therapy versus single agent alone as salvage treatment for AGC patients in this setting.

This meta-analysis is, to the best of our knowledge, the first to compare doublet combination therapy with single agent as second-line treatment for pretreated AGC patients. A total of 1698 pretreated AGC patients are ultimately identified. The pooled results demonstrate that doublet combination agents as second-line treatment for AGC provide substantial benefit for pretreated AGC patients in terms of OS and PFS; it also increases the RR by 57%. In particular, targeted agent plus chemotherapy regimens have been found to reduce the risk of progression and death by 17% and 30%, respectively, while the addition of cytotoxic agents to mono-chemotherapy does not significantly improve OS, PFS, and ORR. Additionally, we also find that taxanes-based doublet therapy is superior to taxanes alone in terms of OS, PFS, and ORR, while no significantly improved survival benefits is observed in CPT-11-based doublet therapy in comparison with CPT-11 alone. However, we should acknowledge that data are immature to make an exact conclusion. In our study, the pretreated regimens for AGC patients are significantly different. For example, 2 RCTs include AGC patients previously treated with S-1 monotherapy; while other studies include AGC patients who refractory to fluoropyrimidine and platinum; in addition, we also include pretreated AGC patients with HER-2 amplification for analysis. All of these would increase the clinical heterogeneity of the meta-analysis, and more evidences from RCTs are needed to appraise the therapeutic effect of doublet combination therapy in this setting.

Safety of systematic treatments is of particular importance in palliative setting in pretreated AGC patients, given the potential negative impact on benefit ratio and quality of life. Finding of our study indicates that there are more incidences of grade 3 and 4 diarrhea, fatigue, myelosuppression toxicities, while equivalent frequencies of nausea and thrombocytopenia are found between single agent and doublet combination therapy.

Our study has the following limitations need to be considered. First, our study is a meta-analysis of published data, and individual patient information is not available. Thus, confounding variables at the patient level, such as co-morbidities and previous treatment, could not be incorporated into the analysis. Second, we include different combination regimens and targeted agents in present study, which might increase the clinical heterogeneity among the included trials, although we perform sub-group analysis according to treatment regimens to detect the potential efficacy difference. Third, the exact combination regimens among included trials are multitudinous; we thus could not answer that which combination regimens would be the best choice. Finally, most of the included studies in our analysis are phase II trials and OS are not always the primary endpoint. Thus, these studies might not be adequately powered for determining a survival difference due to the short follow-up time. Further long-time follow-up studies are still needed.

## CONCLUSIONS

Currently available clinical evidence for pretreated AGC patients indicates that the combination of targeted agent with single cytotoxic agent may be a more efficient regimen for AGC patients due to its significantly survival benefits, but with more frequencies of grade 3 and 4 toxicities in comparison with single agent, while no significantly survival benefits has been observed in doublet cytotoxic chemotherapy groups. Based on our findings, we believe that doublet therapy using targeted agent plus single cytotoxic agent should be offered to fit patients for the second-line treatment of AGC patients. Further studies are recommended to identify patients who will most likely benefit from the appropriate targeted agent-based doublet combination therapy.
